# Intertrigo in Severe Obesity: Clinical Insights and Outcomes With a New Antimicrobial Silver‐Infused Breathable Fabric

**DOI:** 10.1111/jocd.70161

**Published:** 2025-04-30

**Authors:** Franca Pera, Camilla Suman, Mioara Cosma, Silvia Mazza, Amelia Brunani, Raffaella Cancello

**Affiliations:** ^1^ Department of Metabolic and Functional Rehabilitation IRCCS Istituto Auxologico Italiano Italy; ^2^ Department of Metabolic Medicine IRCCS Istituto Auxologico Italiano Italy; ^3^ Obesity Unit and Laboratory of Nutrition and Obesity Research Department of Endocrine and Metabolic Diseases, IRCCS Istituto Auxologico Italiano Milan Italy

**Keywords:** antimicrobial silver, intertrigo, moisture‐wicking fabric, severe obesity, treatment

## Abstract

**Background:**

Intertrigo is often underdiagnosed in obesity.

**Objectives:**

To investigate the incidence of intertrigo in obesity and assess the efficacy of a moisture‐wicking fabric (MWF) with antimicrobial silver.

**Methods:**

Intertrigo symptoms were evaluated in patients with severe obesity, and the effect of MWF application was assessed in a subset of these patients after 5 or 10 days of use.

**Results:**

In inpatients with obesity (*n* = 2.778, mean age 63.9 ± 13.3 years, BMI 43.4 ± 6.8 kg/m^2^), intertrigo lesions were found in 15.9% under the breast (37.4%), abdomen (19.0%), and inguinal area (9.6%). It was more prevalent in women (12.3%), especially among patients older than 65 years (50.9%), with a mean BMI of 46.4 ± 7.3 kg/m^2^, waist circumference of 129.6 ± 15.2 cm, and mean fat mass of 51.1% ± 6.2%. A subset of 40 patients with intertrigo was randomly divided into two groups: one treated with MWF (mean age 57.0 ± 14.2 years, BMI 46.4 ± 8.2 kg/m^2^) and the other with traditional treatment (mean age 62.0 ± 14.1 years, BMI 49.9 ± 7.5 kg/m^2^). The MWF treatment resulted in significantly faster symptom resolution (6.0 ± 2.4 days for MWF vs. 11.0 ± 3.9 days in control group; *p* < 0.001).

**Conclusions:**

We underline the need for intertrigo screening in patients with obesity, especially in those with risk factors, such as female, older, and higher BMI. The use of a novel MWF effectively alleviates symptoms within a short treatment period and should be considered as an optional intertrigo treatment.

## 1 Introduction

The global obesity pandemic has emerged as a significant risk factor for numerous morbid conditions, including type 2 diabetes mellitus, hypertension, dyslipidemia, cardiovascular disease, and certain cancers. Additionally, nearly 50% of patients with obesity exhibit skin comorbidities related to metabolic disorders [1]. Various skin conditions—including keratosis pilaris, plantar hyperkeratosis, acanthosis nigricans, candidiasis, striae distensae, acrocyanosis, atopic dermatitis, lymphedema, hidradenitis suppurativa, cellulitis, and intertriginous eczema—are often underdiagnosed in individuals with obesity [2, 3]. All of these skin manifestations are known to significantly impact quality of life (QoL) [3].

Among these conditions, intertrigo is a chronic inflammatory disorder of skin folds, caused by friction, moisture, and reduced airflow. Initially characterized by erythema [4], intertrigo is often accompanied by maceration, itching, burning, pain, and unpleasant odor [5]. It typically occurs on opposing skin surfaces due to friction, moisture, and limited air circulation [6, 7]. Commonly affected areas include the sub‐mammary region, abdominal folds, groin, axillae, and intergluteal cleft. Less frequently, it can affect periumbilical, interdigital, eyelid, and retro‐auricular regions. Intertrigo is often complicated by infections, most commonly *Candida albicans* and dermatophytes, such as *Trichophyton rubrum*, though bacterial infections with staphylococci, streptococci, Gram‐negative species, and antibiotic‐resistant strains are also prevalent [8]. Recurrence is high in people with obesity, and the associated symptoms can negatively impact QoL through psychological distress [8].

The true incidence and prevalence of intertrigo remain unclear [9], though estimates range from 6% in hospital settings to 17% in nursing homes, peaking at 20% among home care patients [4]. This variation may be due to the strong correlation between intertrigo and patient dependence on caregivers for hygiene maintenance [10]. No studies in Italy have yet examined the incidence or prevalence of intertrigo in patients with obesity.

Obesity is a known risk factor for intertrigo due to increased sweating, pronounced skin folds, impaired skin barrier function, and challenges in maintaining hygiene, exacerbated by reduced mobility [11]. Additional risk factors are represented by clinical complications, such as diabetes, steroid therapy, broad‐spectrum antibiotic use, and acquired immunodeficiency syndrome (caused by HIV) [5]. Also, aging contributes to the intertrigo increased risk, as the skin water content of the stratum corneum decreases to less than 10% in elderly individuals, compromising the cutaneous barrier function. Loffler and Effendy [12] demonstrated that the continuous loss and evaporation of water through the outer layers of the skin predisposes patients with obesity to increased cutaneous maceration risk.

Current treatments for intertrigo aim to eliminate causative factors, such as moisture and friction. When these measures are insufficient, standard treatments focus on managing the symptoms. These include barrier creams, antiseptics, and various fabrics or nonwoven materials placed between skin folds. However, traditional approaches like powders (e.g., corn starch) or gauze often prove ineffective, as they absorb moisture without allowing it to evaporate, thereby promoting skin damage and bacterial growth [6, 13]. Zinc oxide remains a widely used treatment, but its efficacy has not been rigorously studied [14]. Antifungals are often employed for complications, such as candidiasis, although no controlled studies have evaluated their effectiveness [4]. Resolution of intertrigo is frequently prolonged, and symptoms may persist, leading to more severe complications, like mycosis or ulceration [15, 16].

This study aims to evaluate the clinical prevalence of intertrigo in a cohort of severely obese inpatients in Italy. We seek to describe the clinical characteristics of intertrigo in individuals with obesity, with an emphasis on large skin lesions within skin folds. Additionally, in a subset of these patients, we will assess the efficacy and resolution time of a novel polyester fabric coated with a thin polyurethane layer that releases silver ions with antibacterial and antifungal properties via a patented mechanism moisture‐wicking fabric (MWF). Previous studies have shown clinical improvement or resolution within five days of MWF application compared to conventional treatments [9, 17].

## 2 Materials and Methods

The study was conducted at the IRCCS Istituto Auxologico Italiano, Piancavallo, Verbania, Italy. Patients consecutively admitted to the hospital from January to December 2019 for a 4‐week multidisciplinary obesity rehabilitation program (i.e., metabolic, nutritional, and psychological rehabilitation as previously described [18]) were enrolled in the study. The study was approved by the Ethics Committee of Istituto Auxologico Italiano (details available at https://www.auxologico.it/ricerca‐formazione/comitato‐etico) with the approval ID number: code 2019_07_16_5. All procedures were conducted as part of routine hospital care, and each patient provided written informed consent for the use of their data for research purposes upon admission. The research adhered to the principles outlined in the 1964 Helsinki Declaration, its subsequent amendments, and the ethical guidelines set by the institutional and national research.

### 2.1 Patients

**FIGURE 1:**
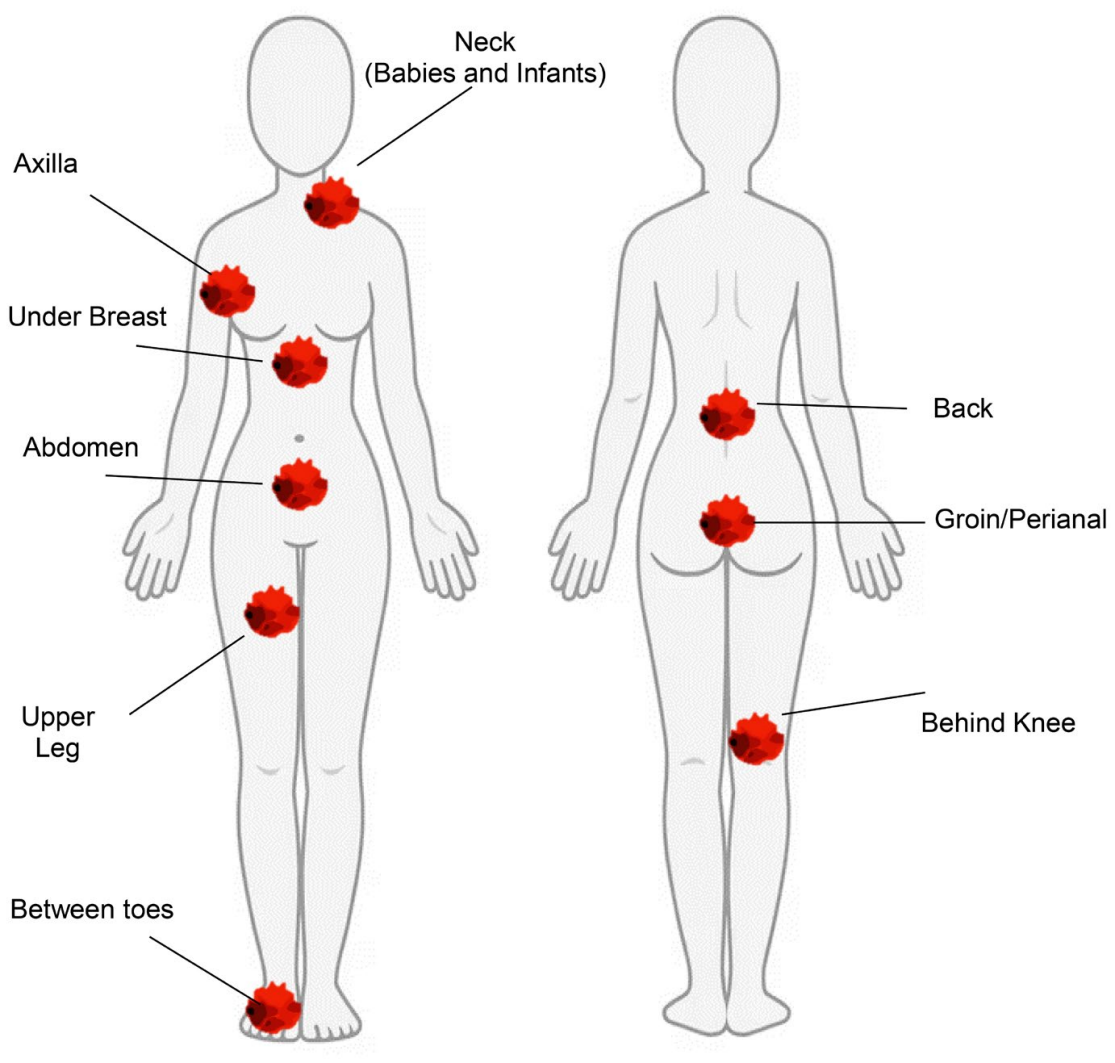
Body representation of common skin areas affected by intertrigo.

**FIGURE 2:**
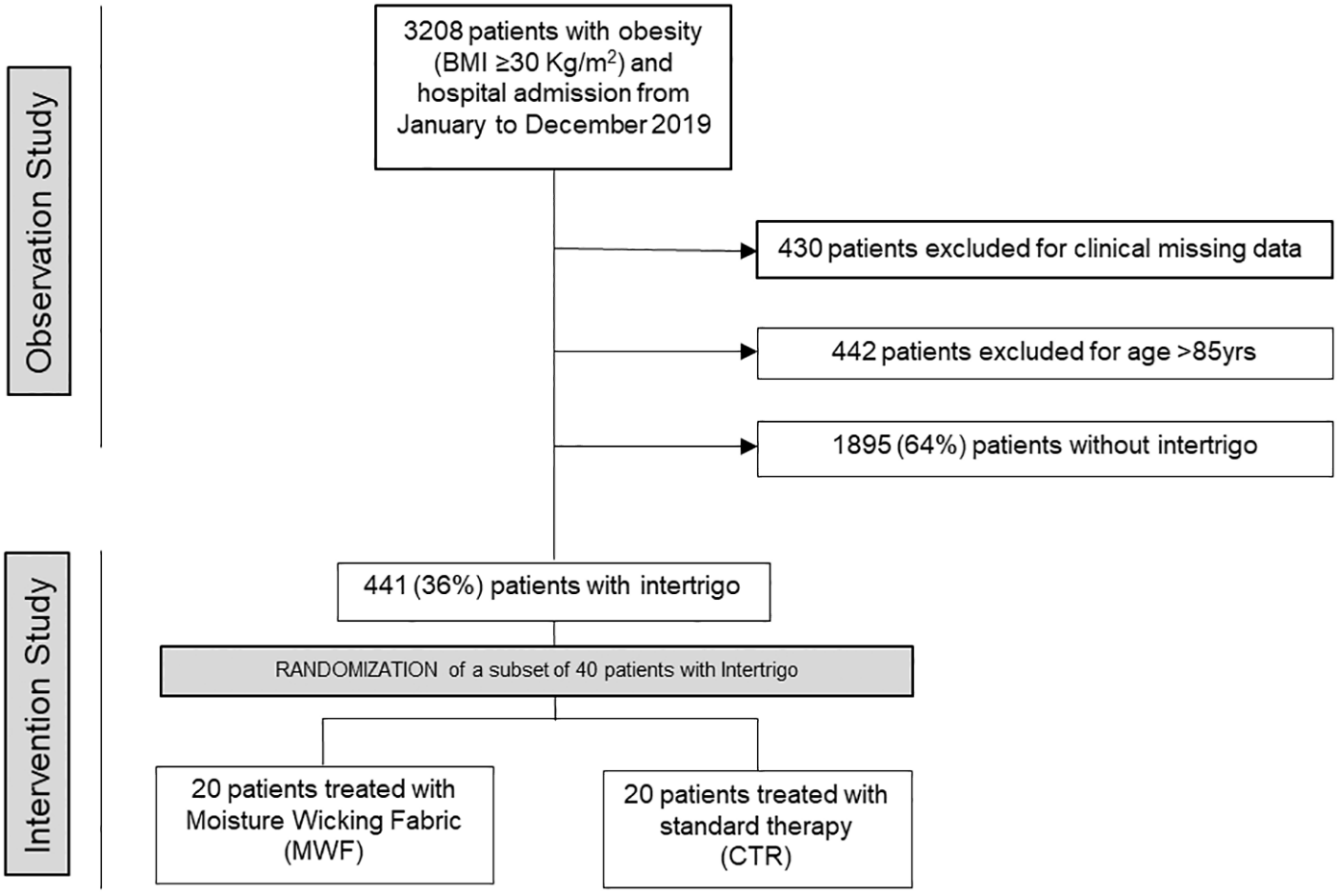
Flow chart of patient enrollment and randomization for treatment.

We enrolled patients of both genders, aged 18–85 years and body mass index (BMI) ≥ 30 kg/m^2^. Clinical data, such as sex, age, presence of comorbidities, and degree of mobility (0 = autonomous in activities of daily living [ADL]; 1 = ADL performed with use of aids for disabled/supervision and 2 = ADL with help by healthcare operator) were collected at admission. Furthermore, during a general examination, the presence of characteristics for intertrigo diagnosis was registered. The lesions were identified for location (neck, axilla, inframammary, dorsal fold, plica abdominal, interdigital, groin, lower limbs) (Figure 1) and symptoms, such as erythema, maceration, itching/burning, de‐epithelization (denudement), satellite lesion, odor, and pain were considered. We analyzed data in patients with or without intertrigo. The summary of the patient's cohort is reported in Figure 2 (flow chart). Patients with age > 85 years, without intertrigo, and with missing data for ADL were excluded.

### 2.2 Anthropometric Parameters and Body Composition

Body weight (kg) and body height (meters) were measured with precision to the nearest 0.1 kg and 0.5 cm, respectively. A mechanical column scale (Scale–Tronix, Wheaton, IL) and a stadiometer (Scale–Tronix, Wheaton, IL) were used for these measurements. BMI was then calculated by dividing the body weight by the square of the height (kg/m^2^). According to WHO [19] the following obesity classes were considered: obesity class I (mild obesity): BMI 30.0–34.9 kg/m^2^; obesity class II (moderate obesity): BMI 35.0–39.9 kg/m^2^; obesity class III (severe or morbid obesity): BMI ≥ 40.0 kg/m^2^. The waist circumference (cm) was measured with nonelastic tape at the level of the umbilicus. Body composition analysis (fat mass, FM, free fat mass, FFM, and muscle mass, MM, expressed as percentage of body weight) was performed by a single‐frequency bioimpedance analyzer (BIA 101, Akern, Pisa, Italy) as previously described [20].

### 2.3 Efficacy Study

A subgroup of 40 patients with an intertrigo diagnosis was randomly assigned to two groups: the first one was treated with a MWF (Tri‐Go), MWF group (*N* = 20), and the second with a classic treatment (local antiseptic/antifungal medications) and used as control (CTR, *N* = 20). The exclusion criteria were a known allergy to silver.

### 2.4 Materials

The MWF used is a soft, conformable, antimicrobial fabric designed to help and manage the conditions associated with intertrigo. This fabric is a silver‐containing moisture‐wicking textile composed of a continuous polyester filament pique textile mesh substrate, buffered with an aqueous suspension of zirconium silver sodium phosphate dibasic and a polyurethane binder. The fabric uses a patented silver antimicrobial complex against microorganisms commonly found in skin folds. The antimicrobial activity can also help to reduce odor. MWF can be applied to an intact or broken skin surface that shows signs of inflammation and/or irritation with symptoms, such as redness, itching, pain, swelling, and warmth. The control (CTR) group was treated with antifungal powder (clotrimazol) and, to reduce moisture, with spray powders containing colloidal silver (Ag) when needed.

### 2.5 Protocol

The clinical data and lesion assessments were conducted at baseline (admission), with the skin lesions examined each day until the 5th day. If treatment needed to be extended, additional assessments were made on the 6th and up to the 10th day. The study duration was based on previous findings that showed improvement in intertrigo symptoms after an average of 5 days of treatment [17]. A 7–10‐day observation time period was chosen to assess not only improvement but also the potential resolution of the lesions. All clinical assessments were performed by the same nursing staff, who were specifically trained to ensure consistent and objective scoring of the skin. Data from the CTR group were similarly collected by the same trained nursing staff, following hospital standardized procedures.

### 2.6 Statistical Analysis

For the frequency analysis, a descriptive approach was used to summarize the characteristics of the enrolled patients and their lesions. For the efficacy analysis, the primary parameter of interest is the potential effect of the treatment. To detect a 70% difference in resolution rates by the fifth day with 95% confidence, a sample of 42 subjects (21 per group) is required, providing a confidence interval with a width of 40% (50%–90%). A 1% success rate in the control group is assumed, in line with the methodology proposed by Teare et al. [21]. The statistical analysis will focus on estimating the proportion of treatment successes in each group by the fifth day, along with a confidence interval for the difference in proportions between groups. Statistical analysis for frequency and efficacy was performed with JMP (Version 16), SAS Institute Inc. https://www.jmp.com SAS Institute Inc. (2021).

## 3 Results

A total of 3208 patients with obesity were admitted and, considering exclusion criteria, 2778 patients were considered for the study: 1627 (59%) were female, with a mean age of 63.9 ± 13.3 years and a mean BMI of 43.4 ± 6.8 kg/m^2^. In relation to age, patients aged ≤ 65 years made up 49.1% of the sample, while those aged ≥ 66 years constituted 50.9%. The prevalence of intertrigo was 15.9%: 12.3% in female and 3.6% in male patients. Patients with intertrigo present a mean age of 66.3 ± 12.0 years, a BMI of 46.4 ± 7.3 kg/m^2^, a waist circumference of 129.6 ± 15.2 cm, an FM percentage of 51.1% ± 6.2%, and a FFM percentage of 48.9% ± 6.2%. Intertrigo was present in 0.8% of patients with Grade I obesity, 1.9% of patients with Grade II obesity, and 13.2% of patients with Grade III obesity.

The skin areas more frequently affected were: 37.4% inframammary, 19.0% abdominal, 9.6% inguinal, and furthermore, 34.0% of patients had two or more areas affected by intertrigo.

**FIGURE 3:**
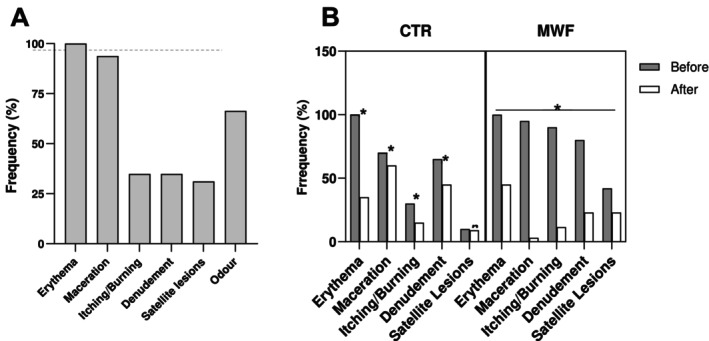
Frequencies of Intertrigo symptoms in studied cohort of patients with obesity, *n* = 441 (panel A); treatment efficacy in the patients randomized for standard treatment (controls, CTR, *n* = 20) and moisture‐wicking fabric treatment (MWF, *N* = 20), (panel B). Levels of statistical significance in the intragroup analysis (*p < 0.05; **p < 0.01; ***p < 0.0001); ns, not significant variation.

Frequency of clinical symptoms of intertrigo at baseline is reported in Figure 3A. Here in detail is the symptom and the relative frequency: erythema (100%), maceration (93.8%), Itching/burning (34.9%), denudement (34.9%), satellite lesions (31.1%) and odor (66.4%).

The more frequently associated diseases registered were diabetes (34.9%) and sleep apnea syndrome (34.5%); 34.2% presented a mobility reduction (ADL > 1).

### 3.1 Efficacy Study

The two groups of patients with intertrigo, randomly assigned to either the standard treatment (CTR, *n* = 20) or the MWF treatment (MWF, *n* = 20), showed no significant differences in age (MWF: 57.0 ± 14.2 years vs. CTR: 62.0 ± 14.1 years; *p* = 0.144) or BMI (MWF: 46.4 ± 8.2 kg/m^2^ vs. CTR: 49.9 ± 7.5 kg/m^2^; *p* = 0.104). None were affected by type 2 diabetes. In both groups, the common areas with intertrigo were sub‐mammary (24.2% in MWF and 27.2% in CTR) and under abdomen (29.6% in MWF and 28.6% in CTR); in 41%, two areas were involved. The frequency of the symptoms was erythema 100%, maceration 84%, Itching/burning 65%, denudement 68%, satellite lesions 31%, and odor 60%. A similar reduction of mobility was present in both groups (eight patients in MWF group, vs. nine patients in CTR group).

In the MWF group, 45% of patients experienced a resolution of intertrigo symptoms within the first 5 days of treatment, compared to only 20% in the control (CTR) group during the same period. By the end of the 5‐day treatment, the MWF group achieved complete resolution of symptoms (100% of patients), whereas the CTR group had only 45% resolution (*p* = 0.0001).

The mean duration to complete resolution was significantly shorter in the MWF group, with an average of 5.8 ± 2.5 days, compared to 10.8 ± 3.9 days in the CTR group (*p* < 0.0001).

Furthermore, the maceration present in 95% of patients at baseline was completely resolved in the MWF group after 5 days, whereas it persisted in 70% of the CTR group, only reducing to 60% after the following 5 days (10th day).

**FIGURE 4:**
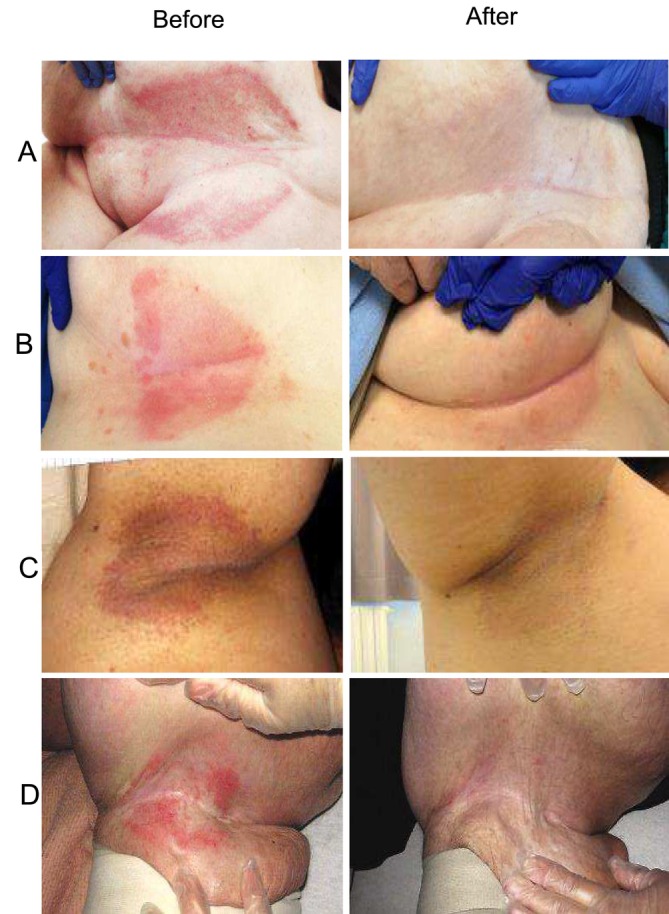
Panel A: A patient with a skin lesion in the lower abdomen caused by intertrigo. The “before” image shows the lesion as a red, inflamed, and irritated area within the skin fold, with visible signs of moisture and mild erosion. The “after” image, taken 20 days later, shows a significant improvement with the lesion healed, the skin smooth, and the redness reduced, indicating recovery after classic treatment (likely involving antifungal or antibacterial medication and proper hygiene). Panel B: A patient with a skin fold lesion under the breasts. The “before” image displays a similar red, irritated lesion with slight maceration and moist appearance. The “after” image, taken 5 days after using moisture‐wicking fabric (MWF), shows a marked reduction in irritation. The skin looks dry and less inflamed, with the fabric visibly reducing friction and moisture, aiding in a more rapid healing process. Panel C: A patient with a skin fold lesion in the axillary (underarm) area. The “before” image shows a reddened and inflamed lesion with visible skin breakdown due to moisture and friction. The “after” image, taken 5 days after MWF treatment, shows a noticeable improvement. The skin in the fold appears less irritated, with reduced redness and inflammation, as the MWF treatment helped keep the area dry and reduced friction. Panel D: A female patients with intertrigo in the right popliteal area. The “before” images shows a red, inflamed, and irritated area within the skin fold, with visible signs of moisture and erosion. The “after” image, taken 5 days after MFW treatment, shows a noticeable improvement.

For the symptom of itching and burning, 90% of patients in the MWF group reported these symptoms at baseline, which decreased to 11.5% following 5 days treatment. In contrast, the CTR group saw a reduction from 30% to 15%. Regarding denudement, it was present in 80% of patients at baseline in the MWF group, which decreased to 23% after the treatment. In comparison, denudement in the CTR group decreased from 65% to 45%. Lastly, satellite lesions were observed in 42% of patients at baseline in the MWF group, reducing to 23% after 5 days. In the CTR group, satellite lesions were present in 10% of patients and reduced to 9% over the same time period (Figure 3B). The effectiveness of standard treatment for intertrigo versus MWF in different body areas is shown in Figure 4.

## 4 Discussion

In this study, we demonstrate that intertrigo is a recurrent condition among individuals with obesity, particularly affecting women, the elderly (≥ 65 years), and those with a BMI greater than 40 kg/m^2^. Our findings underscore the necessity of routinely evaluating intertrigo symptoms during clinical assessments in these patient populations. Importantly, we demonstrate that effective management of this condition can be achieved in a relatively short time frame with the use of a new therapeutic option (MWF).

Previous studies conducted in non‐European populations have emphasized the role of obesity in the development of intertrigo [11, 22]. However, many of these studies did not differentiate between sexes or BMI categories. In contrast, our study involved a large sample of patients with obesity, with an average age lower than that reported in previous literature. In this study, we focused on a population admitted to rehabilitation departments, primarily comprising patients with good mobility and no acute medical issues, making our findings more representative of the general population with obesity. The reported frequency of intertrigo exhibits considerable variability, ranging from 6% to 40% in the US population [4, 23]. This discrepancy can be attributed to several factors, including differences in the populations studied. For instance, a recent European study indicated intertrigo prevalence rates of 2%, 7%, and 10% among hospital admissions, nursing home residents, and home care patients, respectively [10]. In a study by Al‐Mutari [16], a prevalence of 22.2% for intertrigo was found among adult patients with a BMI greater than 25 kg/m^2^. Boza et al. [19] reported a statistically significant association between obesity and skin problems in their study of 76 obese and 73 normal‐weight patients, with 45% of those experiencing intertrigo. These findings suggest that the true prevalence of intertrigo among obese patients may be underestimated, as evidenced by Brown et al. [24], who identified skin issues in 75% of a sample of 100 obese patients through self‐reporting questionnaires. Among these, itching and maceration were particularly common, and 25% of the patients did not seek help, whereas 59% consulted a doctor, and 16% reached out to other healthcare professionals. Also considering the high BMI with large skin folds and increased sweating, the present data show that subjects older and with problems in mobility present a major risk for intertrigo probably due to difficulty in getting dressed and performing hygiene activities as previously reported [10].

Intertrigo, an inflammatory skin condition, is especially common in individuals with obesity due to multiple contributing factors. The primary cause is skin‐on‐skin friction within body folds, which is intensified in obese individuals due to the greater number and depth of these folds. Additionally, obesity often results in poor air circulation within skin folds, trapping moisture and creating an environment conducive to inflammation and infection. This warm, moist environment frequently leads to secondary bacterial or fungal infections. At present, a critical clinical challenge is the absence of specific assessment tools that facilitate the identification (and the staging) of intertrigo and its associated risk factors, such as diabetes, obesity, and previous medication use. The description of the major representative symptom (operator dependent), erythema and maceration, might help to recognize intertrigo in the early phase before the appearance of the signs of erosion (satellite lesion) that are less frequently seen in obesity.

Topical corticosteroids, such as 1% hydrocortisone cream, are frequently used to reduce inflammation and have demonstrated high remission rates. Barrier creams and moisturizers also play a crucial role, helping to protect the skin and maintain moisture balance, with studies showing moisturizers improve skin barrier function and symptom relief more effectively than powders. For cases involving fungal infections, topical antifungals, like miconazole, clotrimazole, and nystatin are recommended, especially for candida intertrigo. To control moisture, drying agents, such as absorptive powders (e.g., corn starch) and drying gels containing miconazole nitrate, help minimize irritation [5]. In cases with secondary bacterial infections, antibacterial treatments are prescribed based on the pathogens involved, such as mupirocin for streptococcal infections or erythromycin for Corynebacterium infections. Lifestyle modifications are also essential, with patients encouraged to wear light, nonrestrictive, absorbent clothing, avoid wool and synthetic fibers, and practice good hygiene, including thoroughly drying skin folds after washing [9]. However, many patients endure prolonged treatments without effective relief, as underlying risk factors remain unaddressed. As noted in a recent “consensus clinical expert opinion” for managing intertrigo [25], moisture‐wicking textiles (MWF) are a recommended therapeutic option for both mild and severe lesions. Our experience suggests that the application of MWF can significantly alleviate intertrigo symptoms in various anatomical locations within just five days, leading to the resolution of symptoms in a substantial proportion of patients. In contrast, traditional management options, such as drying agents, barrier creams, topical antifungals, and absorptive material, demonstrated less efficacy within the same timeframe. It is suggested that the efficacy of MWF is probably due to minimizing skin friction and reducing moisture facilitating the evaporation; these effects produce skin fold dry and the antimicrobial silver probably prevents possible infections [17].

Effective management and prevention of intertrigo involve implementing clear skin care protocols, including dedicated skin fold management, to improve patient outcomes. Additionally, promoting weight management and addressing underlying conditions can help reduce both the occurrence and recurrence of intertrigo. Together, these measures can significantly decrease the incidence and severity of intertrigo in individuals with obesity [8].

This study has certain limitations. One potential limitation is the lack of specific microbiological analyses for diagnosing infection in moisture‐associated skin damage, which is essential in cases of severe ulceration. Additionally, specific predictors for intertrigo, such as the impact of systemic treatments, were not evaluated.

In conclusion, while we advocate for the use of MWF, it is important to promote lifestyle modifications to mitigate risk factors associated with intertrigo. A comprehensive skin assessment should be included for the management of all patients with obesity, along with a well‐structured prevention plan tailored by nursing staff at the time of hospital admission. Additionally, patient education should be prioritized throughout recovery to foster awareness and proactive management of skin health.

## Author Contributions

F.P.: conception and design; C.S., M.C., and S.M.: acquisition of data; F.P., A.B., and R.C.: analysis and interpretation of data; A.B. and R.C.: drafting the manuscript. Each author approved the final version.

## Ethics Statement

The study was approved by the Ethics Committee of the Italian Auxological Institute (code 2019_07_16_5).

## Consent

Each patient signed an informed consent form.

## Conflicts of Interest

The authors declare no conflicts of interest.

## Data Availability

The data underlying this article will be shared on reasonable request to the corresponding author. Each author approved the final version.
